# Correction to Tight control and early disease intervention increase the rates of transmural remission in Crohn's disease

**DOI:** 10.1002/ueg2.12680

**Published:** 2024-10-10

[Fernandes SR, Bernardo S, Saraiva S, Gonçalves AR, Moura Santos P, Valente A, Correia LA, Cortez‐Pinto H, Magro F. Tight control using fecal calprotectin and early disease intervention increase the rates of transmural remission in Crohn's disease. United European Gastroenterol J. 2024 May; 12(4):451–458.]

In the section “Intervention in early disease,” the percentages “48.6% versus 12.0%, *p* = 0.005” were incorrect. This should have read “50.0% versus 12.0%, *p* = 0.003.” The correction also applies to Figure 2. The updated Figure 2 appears below.


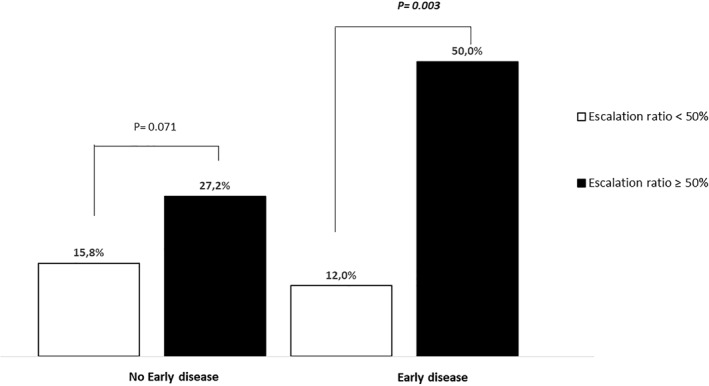


We apologize for this error.

